# Association of Hypertrophic Obstructive Cardiomyopathy with Rheumatoid Arthritis

**DOI:** 10.7759/cureus.2028

**Published:** 2018-01-05

**Authors:** Mustafa Dawood, Noman Lateef, Abubakar Tauseef, Janki Patel

**Affiliations:** 1 General Internal Medicine, Greater Baltimore Medical Center; 2 Department of Medicine, Dow University of Health Sciences (DUHS), Karachi, Pakistan

**Keywords:** hypertrophic obstructive cardiomyopathy, autoimmune diseases, rheumatoid arthritis, hla-dr antigens, echocardiography

## Abstract

Our article refers to a 50-year-old woman with previously diagnosed rheumatoid arthritis (RA) who presented with symptoms of dyspnea on exertion and dizziness. An echocardiogram revealed a 17-mm asymmetric interventricular septum hypertrophy and systolic anterior motion of the anterior mitral valve leaflet. Association of hypertrophic obstructive cardiomyopathy (HCM) with connective tissue diseases has been well documented. For RA the human leukocyte antigen (HLA) system, particularly HLA-DR4, may possibly be a link between the two entities, as it is associated with both RA and HCM. Therefore, a patient with RA and suggestive history should be investigated for cardiac involvement. Further studies are needed to elucidate a more accurate association between the above diseases.

## Introduction

Rheumatoid arthritis (RA) is a multisystem disease that most commonly affects the joints but it can also damage a wide variety of body systems, including the skin, eyes, lungs, heart, and blood vessels. Among patients with RA, heart involvement is a frequent cause of death. Hypertrophic obstructive cardiomyopathy (HCM) is one of the least common cardiac manifestations occurring in a patient with RA [1-2]. It is usually familial in most cases but can be sporadic as well, with an autosomal dominant inheritance [3].

The case presented here is of a patient with RA who presented with syncope on exertion and subsequently diagnosed with HCM on echocardiography. The only similar case was reported eight years back in 2009, in which a patient with diagnosed RA presented with a complaint of dyspnea on exertion and was eventually found to have HCM.

## Case presentation

A 50-year-old female with a known history of RA was admitted to Greater Baltimore Medical Center (Maryland, USA) due to symptoms of syncope on exertion and dizziness for two weeks. RA was diagnosed thirty years ago on the basis of clinical and laboratory findings of symmetrical polyarthralgias and positive RA factor and anti-cyclic citrullinated peptide (CCP) antibody respectively.The patient was treated with methotrexate for the past ten years, and four months before admission corticosteroids (5 mg daily prednisone) were started because of pleuritis. No recent changes were made to the treatment regimen.

On physical examination, her heart rate was 52 beats/min and her blood pressure was 91/45 mmHg. On auscultation, a non-radiating ejection systolic murmur, best heard on the left parasternal border, was revealed. Laboratory findings showed normocytic normochromic anemia (hemoglobin (Hb) 12.2 g/dL, hematocrit 34.7%, mean corpuscular volume (MCV) 94 fL/cell), white blood cell count 13.09 x 10^9^/L, platelet count 194x 10^9^/L, blood urea nitrogen (BUN) 11 mg/dL, creatinine 0.8 mg/dL, C-reactive protein (CRP) 4.68 mg/L, erythrocyte sedimentation rate (ESR) 51 mm/hr, thyroid stimulating hormone (TSH), adrenocorticotropic hormone (ACTH) stimulation tests were all within normal limits. She was seropositive for the rheumatoid factor with a titer of 1:10.

**Figure 1:**
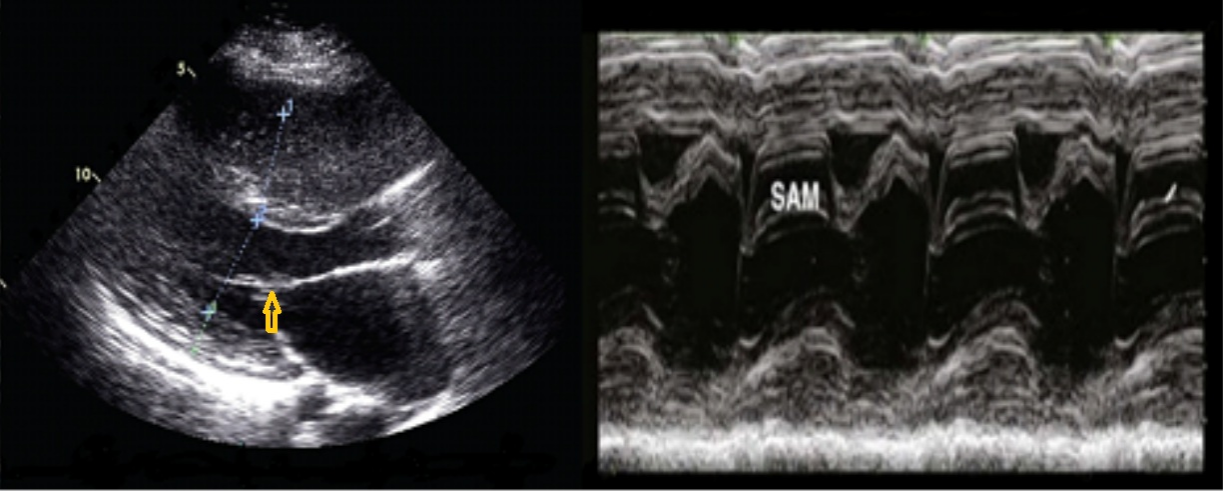
2-D Echocardiography Parasternal long-axis view of the patient’s left ventricle showing asymmetric interventricular septum hypertrophy *(left)*, and M-mode showing the systolic anterior motion (SAM) of the mitral valve leaflet *(right)*

A resting electrocardiogram demonstrated sinus bradycardia. Transthoracic echocardiography showed left ventricular wall thickness with asymmetric interventricular septum hypertrophy (17 mm), abnormal diastolic filling pattern and systolic anterior motion (SAM) of the anterior mitral valve leaflet [Figure 1].These findings are consistent with hypertrophic obstructive cardiomyopathy. The ejection fraction was 70%.There was no family history of sudden death or HCM. The patient received fludrocortisone 0.1 mg daily as well as metoprolol tartrate 25 mg twice daily. Subsequently, her symptoms improved and follow-up with cardiology was scheduled.

## Discussion

RA is a common chronic autoimmune disease involving many organ systems. It primarily affects the joints but also frequently involves the heart, eye, skin, lung, renal, nervous, and gastrointestinal systems. Of these, heart involvement is the leading cause of death, where it manifests as lesions in the pericardium, myocardium, and endocardium, comprising coronary arteries, valvular tissue and the conduction system [1-2].

HCM is characterized by hypertrophy and fibrosis occurring without any known etiology. The primary abnormality responsible for HCM is a genetic defect. The pattern of inheritance is autosomal dominant, which means that only one of the alleles is defective [3]. In familial HCM, 10 gene abnormalities have been discovered in the sarcomere, i.e., the genes of β-cardiac myosin heavy chain, troponin T, α-tropomyosin, myosin-binding protein C, essential or regulatory myosin light chain, troponin I and C, α-cardiac actin and titin [4].

Previous reports have found that the transmission of HCM seems to be related to the human leukocyte antigen (HLA). However, other studies could not validate this association. HCM can be divided into two subtypes: a sporadic form which bears no link to HLA antigens, and a familial form linked to the HLA system, which may be related to the obstructive type. The HLA system may possibly be a link between the two entities, as the phenotype HLA-DR4 is associated with RA as well as HCM both [5].

Rheumatic disease can be viewed as a ‘natural experiment’ in the interplay between chronic inflammation and heart disease, which could elucidate the fundamental mechanisms by which inflammation accelerates development of atherosclerosis and heart disease [6].

It should be mentioned that the patient had no history of rheumatic heart disease, hypertension, valvular disease or renal involvement, conditions often associated with left ventricular hypertrophy. The steroids were also used only for short duration and in low dose, therefore it is unlikely to be the cause of left ventricular hypertrophy in this case. 

A comprehensive review of the literature shows very few reported cases that associated RA with HCM. Moyssakis, et al. reported a case of a 60-year-old woman with RA, who presented with dyspnea on exertion and was subsequently diagnosed with HCM on echocardiography [7]. The second case was reported by Vocian, et al. of a female patient who was diagnosed with Felty syndrome, while her echocardiogram concomitantly revealed HCM [8].  Apart from RA, other connective tissue diseases like Fabry disease, systemic sclerosis, systemic lupus erythematosus, and polyarteritis nodosa, are associated with HCM as well [9-10]. 

## Conclusions

All of these studies put an impression that there is some association between connective tissue disease and HCM. Hence, a patient with RA and a suggestive history should be investigated for cardiac involvement. Further studies need to be conducted on the clinical and genetic level in order to determine the accuracy of this relationship, so that we may efficiently predict and manage the associated entities. 

## References

[1] Lebowitz WB (1963). The heart in rheumatoid arthritis (rheumatoid disease). A clinical and pathological study of sixty-two cases. Ann Intern Med.

[2] Guedes C, Bianchi-Fior P, Cormier B, Barthelemy B, Rat AC, Boissier MC (2001). Cardiac manifestations of rheumatoid arthritis: a case-control transesophageal echocardiography study in 30 patients. Arthritis Rheum.

[3] Roberts R, Sigwart U (2005). Current concepts of the pathogenesis and treatment of hypertrophic cardiomyopathy. Circulation.

[4] Takeda N (2003). Cardiomyopathy: molecular and immunological aspects (review). Int J Mol Med.

[5] Roudier J (2000). Association of MHC and rheumatoid arthritis. Association of RA with HLA-DR4: the role of repertoire selection. Arthritis Res.

[6] Crowson CS, Liao KP, Davis JM 3rd (2013). Rheumatoid arthritis and cardiovascular disease. Am Heart J.

[7] Moyssakis I, Lionakis N, Votteas V (2009). Hypertrophic obstructive cardiomyopathy in rheumatoid arthtritis - coincidence or association? A case report. Exp Clin Cardiol.

[8] Voncina D, Rozman B, Cijan A (1984). Felty's and Sjögren's syndromes and hypertrophic obstructive cardiomyopathy (case report). Z Rheumatol.

[9] Chimenti C, Pieroni M, Morgante E (2004). Prevalence of Fabry disease in female patients with late-onset hypertrophic cardiomyopathy. Circulation.

[10] Anastasiadis GP, Moyssakis I, Boki K, Kyriakidis M (2001). Hypertrophic cardiomyopathy in systemic lupus erythematosus. Mayo Clin Proc.

